# Osteopontin selectively regulates p70S6K/mTOR phosphorylation leading to NF-κB dependent AP-1-mediated ICAM-1 expression in breast cancer cells

**DOI:** 10.1186/1476-4598-9-101

**Published:** 2010-05-07

**Authors:** Mansoor Ahmed, Gopal C Kundu

**Affiliations:** 1 National Center for Cell Science (NCCS), NCCS Complex, Pune 411 007, India

## Abstract

**Background:**

Breast cancer is one of the most frequently diagnosed cancer and accounts for over 400,000 deaths each year worldwide. It causes premature death in women, despite progress in early detection, treatment, and advances in understanding the molecular basis of the disease. Therefore, it is important to understand the in depth mechanism of tumor progression and develop new strategies for the treatment of breast cancer. Thus, this study is aimed at gaining an insight into the molecular mechanism by which osteopontin (OPN), a member of SIBLING (Small Integrin Binding LIgand N-linked Glycoprotein) family of protein regulates tumor progression through activation of various transcription factors and expression of their downstream effector gene(s) in breast cancer.

**Results:**

In this study, we report that purified native OPN induces ICAM-1 expression in breast cancer cells. The data revealed that OPN induces NF-κB activation and NF-κB dependent ICAM-1 expression. We also observed that OPN-induced NF-κB further controls AP-1 transactivation, suggesting that there is cross talk between NF-κB and AP-1 which is unidirectional towards AP-1 that in turn regulates ICAM-1 expression in these cells. We also delineated the role of mTOR and p70S6 kinase in OPN-induced ICAM-1 expression. The study suggests that inhibition of mTOR by rapamycin augments whereas overexpression of mTOR/p70S6 kinase inhibits OPN-induced ICAM-1 expression. Moreover, overexpression of mTOR inhibits OPN-induced NF-κB and AP-1-DNA binding and transcriptional activity. However, rapamycin further enhanced these OPN-induced effects. We also report that OPN induces p70S6 kinase phosphorylation at Thr-421/Ser-424, but not at Thr-389 or Ser-371 and mTOR phosphorylation at Ser-2448. Overexpression of mTOR has no effect in regulation of OPN-induced phosphorylation of p70S6 kinase at Thr-421/Ser-424. Inhibition of mTOR by rapamycin attenuates Ser-371 phosphorylation but does not have any effect on Thr-389 and Thr-421/Ser-424 phosphorylation of p70S6 kinase. However, OPN-induced phosphorylation of p70S6 kinase at Thr-421/Ser-424 is being controlled by MEK/ERK pathway.

**Conclusion:**

These results suggest that blocking of OPN-induced ICAM-1 expression through mTOR/p70S6 kinase signaling pathway may be an important therapeutic strategy for the treatment of breast cancer.

## Introduction

Breast cancer is one of the major causes of death among all other cancers in women globally. It emerges through a multi-step process starting from hyperplasia to premalignant change, in situ carcinoma, and invasive breast cancer [1-3]. Osteopontin (OPN), a calcified ECM associated non collagenous, sialic acid rich, glycosylated phosphoprotein is secreted by majority of the normal and transformed cells [4]. OPN isolated from different cellular sources, have molecular weight ranging from 44 kDa to 75 kDa due to differences in the post translational modifications [4]. Many highly metastatic transformed cells synthesize high level of OPN than their normal counter parts. Recently it has been reported that OPN plays crucial role in cell migration and invasion by interacting with its receptor α_v_β_3 _integrin by inducing the expression of urokinase plasminogen activator (uPA) and activation of matrix metalloproteinases (MMPs) in various cancer cells [5-9]. Increased level of OPN has been reported in number of human carcinomas, glioblastoma, and osteosarcoma and considered as a lead marker during breast cancer progression [10].

The mammalian target of rapamycin (mTOR), (also known as FRAP/RAFT/RAPT), a member of the phosphatidylinositol 3-kinase-related kinase (PIKK) super family, is consisted of 2549 amino acids that are grouped into highly conserved domains [11,12]. Previous reports have indicated that mTOR acts as a downstream molecule in the PI 3-kinase/Akt signaling pathway. It is an evolutionarily conserved 289-kDa serine-threonine kinase that regulates both cell growth and cell-cycle progression through its ability to integrate signals in response to nutrients and growth factors. mTOR is phosphorylated at Ser-2448 via the PI 3-kinase/Akt pathway and autophosphorylates at Ser-2481 [12-14]. mTOR initiates translation by activating the p70S6 kinase (S6K1) and by inhibiting the eIF4E inhibitor, 4E-BP1. By targeting mTOR, the immunosuppressant and anti-proliferative agent, rapamycin inhibits the signals required for cell-cycle progression, cell growth and proliferation in both normal and malignant cells. Interaction of FKBP12 (immunophilin FK506 binding protein 12 kDa)-rapamycin complex with mTOR inhibits its function and leads to dephosphorylation and inactivation of p70S6 kinase [14-16]. As a result, mTOR may act as an important target for regulation of cancer progression. Activation of p70S6 kinase involves a complex interplay among sequential phosphorylation events, which occur within distinct intramolecular regulatory domains. Phosphorylation of p70S6 kinase at Thr-421/Ser-424 exists in the autoinhibitory domain of carboxyl terminal, Thr-229 in activation loop, Thr-389 and Ser-371 in the linker domain, are very important for the activation of p70S6 kinase [17,18]. The phosphorylation of p70S6 kinase at Thr-421/Ser-424 leads the phosphorylation of other regulatory site by release of pseudosubstrate suppression in the autoinhibitory domain leading to modulation of the kinase activity [19,20]. However, the mechanism by which OPN regulates mTOR/p70S6 kinase activation in breast cancer cells is not well defined.

Nuclear factor κB (NF-κB) and activator protein-1 (AP-1) are key transcription factors that regulate the expression of many genes involved in inflammation, apoptosis, and oncogenesis [21]. Many reports have demonstrated that these transcription factors are thought to be regulated by the same intracellular signal transduction pathway. The activity of NF-κB is regulated by its interaction with the family of NF-κB inhibitor known as IκB, which results in the formation of inactive NF-κB-IκB complex in the cytoplasm. In response to various stimuli, IκB kinase (IKK) phosphorylates IκB. The subsequent proteosome mediated degradation of IκB expose the nuclear localization signal of NF-κB, thus allowing its translocation to the nucleus where it activates the transcription of various target genes including ICAM-1 [21-23]. AP-1 is a group of basic leucine zipper (bZIP) transcription factor consisting of the Fos and Jun families. Extracellular stimuli and growth factor stimulate MAPK pathways which play important role in regulation of transcription factor AP-1, as its activation leads to the induction of c-Fos which associate to c-Jun to form an AP-1 heteromeric complex that can promote target gene expression [21,24]. Our previous results showed that OPN induces cell motility, tumor growth and angiogenesis through NF-κB and AP-1 dependent activation and expressions of MMP-2, -9, uPA, Cox-2 and VEGF in various cancer cells [10,25]. However, the signaling pathways by which OPN controls NF-κB and AP-1 activation and whether there is any cross talk between NF-κB and AP-1 in regulation of ICAM-1 expression is not well understood.

Cell adhesion is a crucial step for normal development and maintenance of tissues and organs. Cell-cell and cell-matrix interaction are mediated by dynamic interaction between various cell surface receptors which play important role in regulation of cancer progression. Based on the structure and functions, adhesion molecules are classified into four major categories: integrins, cadherins, selectins and immunoglobulins superfamilies. The various cell adhesion molecules also function as receptors for various ligands thereby control signal transduction pathways which ultimately regulate cell adhesion, proliferation, migration and differentiation [26,27]. Intercellular adhesion molecule-1 (ICAM-1), also known as CD54 is a cell surface glycoprotein that belongs to the immunoglobin superfamily of adhesion molecules. It is expressed in breast cancer tissues. The process of tumor growth involves alterations in expression of adhesion molecules that may lead to destruction of tissue architecture leading to metastasis [28-30]. The mechanisms by which OPN regulates ICAM-1 expression through mTOR/p70S6 kinase and NF-κB/AP-1 pathways are not defined well.

In summary, we report that OPN regulates NF-κB mediated ICAM-1 expression in breast cancer cells. OPN-induced NF-κB controls unidirectional AP-1 activation, indicating a cross talk between NF-κB and AP-1 which in turn regulates ICAM-1 expression in these cells. We also investigated the role of mTOR and p70S6 kinase in OPN-induced ICAM-1 expression. Our results revealed that both mTOR and p70S6 kinase are involved in OPN-induced ICAM-1 expression. Overexpression of mTOR inhibits OPN-induced NF-κB and AP-1 DNA-binding and transcriptional activity. OPN selectively induces p70S6 kinase phosphorylation at Thr-421/Ser-424. However, overexpression of mTOR has no effect on regulation of OPN-induced Thr-421/Ser-424 phosphorylation. Inhibition of mTOR by rapamycin attenuates Ser-371 phosphorylation of p70S6 kinase. Moreover, OPN-induced phosphorylation of p70S6 kinase at Thr-421/Ser-424 is being controlled by MEK/ERK pathway. Thus, blocking OPN-induced ICAM-1 expression through mTOR and p70S6 kinase pathway may act as important target for the control of breast cancer.

## Materials and methods

### Antibodies, Reagents, and Cell Lines

Rabbit polyclonal anti-ICAM-1, goat polyclonal anti-actin, mouse monoclonal anti-p70S6 kinase, mouse anti-p-ERK1/2 and rabbit anti-ERK2 antibodies were purchased from Santa Cruz Biotechnology. Rabbit anti-p-mTOR antibody was purchased from R&D Systems. Rabbit anti-mTOR, anti-p-p70S6K antibodies and rapamycin were purchased from Cell Signaling Technology. U0126 was obtained from Calbiochem. Anti-human α_v_β_3 _integrin (MAB1976) blocking antibody was from Chemicon International. Lipofectamine 2000 was purchased from Invitrogen. AP-1 consensus oligonucleotide was purchased from Santa Cruz and NF-κB consensus oligonucleotide was purchased from Promega. The [γ-32p] ATP was purchased from Board of Radiation and Isotope Technology (Hyderabad, India). The human OPN was purified from milk as described previously [7] with minor modifications and used throughout this study. The low invasive (MCF-7) and highly invasive (MDA-MB-468) breast cancer cells were purchased from American Type Culture Collection (Manassas, VA). These cells were cultured in Dulbecco's modified Eagle's medium (DMEM) and leibovitz's L-15 supplemented with 10% fetal calf serum, 100 units/ml penicillin, 100 μg/ml streptomycin and 2 mM glutamine in a humidified atmosphere at 37°C.

### Plasmids and DNA Transfection

The wild type and rapamycin resistant mTOR in pIRES-GFP expression vector were a generous gift from Dr. Rok Humar (University Hospital Basel, Switzerland). The wild type and rapamycin resistant HA-S6K1 (p70S6 kinase 1) in pRK7 expression vector were kind gift from Dr. John Blenis (Harvard Medical School, Boston). The super-repressor form of IκBα fused downstream to a FLAG epitope in an expression vector (pCMV4) was a gift from Dr. Dean Ballard (Vanderbilt School of Medicine, Nashville, TN). The wild type pCEFL-GFP-c-Fos was a kind gift from Dr. Omar A Coso (Laboratorio de Fisiologia y Biologia Molecular. Facultad de Ciencias Exactas y Naturales. Universidad de Buenos Aires. IFIBYNE-CONICET. ARGENTINA). The dominant negative c-Fos (also called as A-Fos) in pCMV500 expression vector was a kind gift from Dr. Nicole Darack (NCI, NIH). The wild type c-Jun in pRJB10B expression vector and dominant negative c-Jun in pELFIN expression vector were kind gifts from Dr. Jalam (Ochsner Clinic foundation, New Orleans, LA). The ICAM-1 Luc (containing 1393 bp of ICAM-1 promoter) construct was a kind gift from Dr. Arshad Rahman (University of Rochester School of Medicine, Rochester, New York). The MCF-7 cells were transiently transfected with cDNA using Lipofectamine 2000 according to manufacturer's instructions (Invitrogen). Transfected cells were used for ICAM-1 expression, NF-κB and AP-1-DNA binding, NF-κB, AP-1 and ICAM-1 luciferase assays and p70S6 kinase phosphorylation studies.

### Western Blot Analysis

For ICAM-1 expression, MCF-7 and MDA-MB-468 cells were treated with OPN in a time and dose dependent manner. In separate experiments, MCF-7 cells were either transfected with various cDNA constructs or pretreated with 20 nM rapamycin for 1 h and then treated with 0.5 μM OPN and level of ICAM-1 was detected. For p70S6 kinase and mTOR phosphorylations, cells were treated with 0.5 μM OPN for 0-120 min. In other experiments, the cells were either transfected with mTOR constructs or pretreated with 20 nM rapamycin or 0-500 μM U0126 for 1 h and then treated with 0.5 μM OPN. The cells were lysed in lysis buffer (50 mM Tris-HCl (pH 7.5), 150 mM NaCl, 1% Nonidet P-40, 0.5% sodium deoxycholate, 5 mM dithiothreitol and 1 mM phenylmethylsulfonyl fluoride) and the protein concentrations in cleared supernatants were measured by using Bio-Rad protein assay. The supernatant (lysates) containing equal amount of total proteins (30 μg) were resolved by SDS-PAGE and electrotransferred from gel to nitrocellulose membranes. The membranes were incubated with anti-p-p70S6K, anti-p-mTOR, anti-p-ERK1/2 or anti-ICAM-1 antibodies and further incubated with horseradish peroxidase-conjugated IgG and detected by luminol reagent (Santa Cruz) according to the manufacturer's instruction. The same blots were re-probed with anti-actin or non phospho antibodies of respective molecules and detected.

### Nuclear Extracts and Electrophoretic Mobility Shift Assay (EMSA)

The NF-κB and AP-1 EMSA were performed as described earlier [8]. Briefly, MCF-7 cells were treated with 0.5 μM OPN for 0-240 min at 37°C. In another experiments, cells were transfected with mTOR, treated with 20 nM rapamycin for 1 h and then with 0.5 μM OPN for 30 min. In separate experiments, cells were transfected with wt c-Jun, dominant negative c-Jun, c-Fos and A-Fos cDNAs and then treated with 0.5 μM OPN for 30 min. Cells were scraped, washed with phosphate-buffered saline (pH 7.4) and resuspended in hypotonic buffer (10 mM Hepes, (pH 7.9), 1.5 mM MgCl_2_, 10 mM KCl, 0.2 mM phenylmethylsulfonyl fluoride, and 0.5 mM dithiothreitol), and allowed to swell on ice for 10 min. Cells were homogenized in a Dounce homogenizer. The nuclei were separated by spinning at 3300 ×g for 15 min at 4°C. The nuclear pellet was extracted in nuclear extraction buffer (20 mM Hepes, (pH 7.9), 0.4 M NaCl, 1.5 mM MgCl_2_, 0.2 mM EDTA, 2.5% glycerol, 0.5 mM phenylmethylsulfonyl fluoride and 0.5 mM DTT) for 30 min on ice, and centrifuged at 12,000 ×g for 15 min at 4°C. The supernatant was used as nuclear extract. The protein concentrations in the supernatant of nuclear extracts were measured by Bio-Rad protein assay. The nuclear extracts (10 μg) were incubated with 16 fmol of ^32^P labeled double stranded NF-κB (5'-AGT TGA GGG GAC TTT CCC AGG C-3') and AP-1 (5'-CGC TTG ATG ACT CAG CCG GAA-3') consensus oligonucleotide in binding buffer (250 mM Hepes, (pH 7.9), 5 mM EDTA, 5 mM DTT, 10% Nonidet P-40, 50% glycerol, and 500 mM NaCl) containing 1 μg of salmon sperm DNA. The DNA-protein complex was resolved on a native polyacrylamide gel, and analyzed by autoradiography.

### Luciferase Reporter Gene Assay

The luciferase reporter gene assay was done as described [6]. Briefly, MCF-7 cells were transfected with ICAM-1-Luc using Lipofectamine 2000 and treated with 20 nM rapamycin for 1 h and then with 0.5 μM OPN. In separate experiments, MCF-7 cells were transfected with NF-κB-Luc or AP-1-Luc and then either cotransfected with wt mTOR, rapamycin resistance mTOR or pretreated with 20 nM rapamycin for 1 h and then treated with OPN. In other experiments, cells were transfected with AP-1-Luc and cotransfected with IκBα super repressor or treated with 10 μg/ml anti-α_v_β_3 _integrin blocking antibody for 3 h and then treated with OPN. In another experiments, cells were transfected with NF-κB-Luc and then either cotransfected with wt and dominant negative c-Jun, c-Fos or A-Fos and then treated with OPN. The transfection efficiency was normalized by cotransfecting the cells with pRL vector (Promega) containing a full length Renilla luciferase gene under the control of constitutively active promoter. The cells were harvested in passive lysis buffer and the luciferase activity was measured using the dual luciferase assay system (Promega) according to the manufacturer instruction. Changes in activity with respect to control were calculated.

## Results

### OPN-induces ICAM-1 expression in breast cancer cells

**Figure 1:**
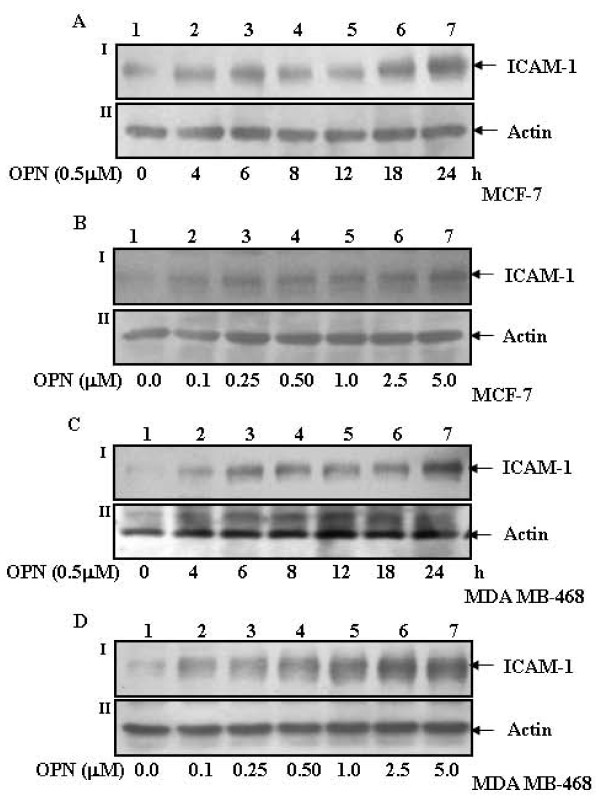
**OPN-induces ICAM-1 expression in breast cancer cells. A and C**. MCF-7 or MDA-MB-468 cells were treated with 0.5 μM OPN in serum free medium for 0-24 h. **B and D**. In a separate experiments, MCF-7 or MDA-MB-468 cells were treated with various concentrations of OPN (0-5.0 μM) for 24 h. Cell lysates containing equal amount of total proteins were analyzed by western blot using anti-ICAM-1 antibody. Actin was used as loading control.

To determine whether OPN-induces ICAM-1 expression, MCF-7 or MDA-MB-468 cells were treated with 0.5 μM OPN for 0-24 h and the expression of ICAM-1 in cell lysates were detected by western blot. The data indicated that OPN induces ICAM-1 expression in time dependent manner in these cells (Fig. 1A &1C, upper panels). The dose dependent response of OPN (0-5.0 μM) on ICAM-1 expression was also detected in these cells and the results showed that the expression of ICAM-1 increases in dose dependent manner and 0.5 μM OPN exhibit significantly high level of ICAM-1 expression as compared to untreated cells (Fig. 1B &1D, upper panels). Actin was used as loading control (Fig. 1A-D, lower panels).

### Both mTOR and p70S6 kinase suppress OPN-induced NF-κB and AP-1 mediated ICAM-1 expression

**Figure 2:**
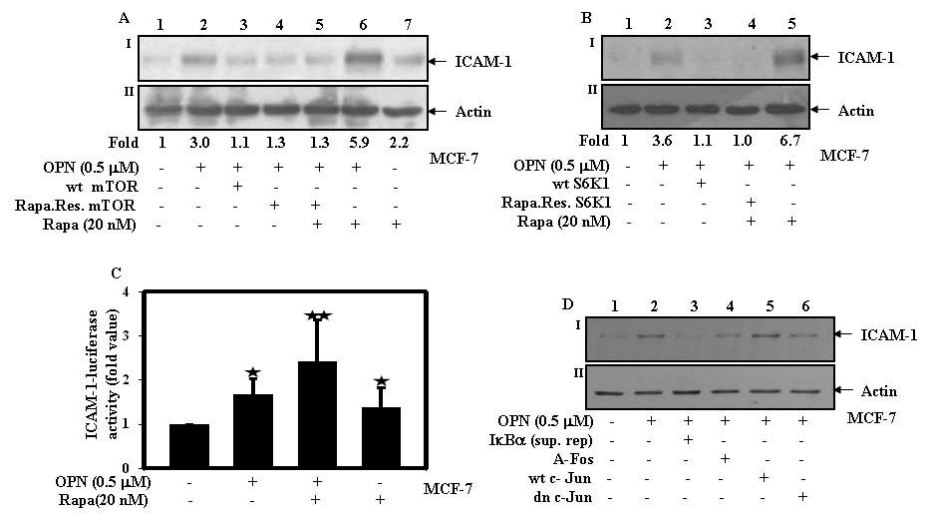
**Both mTOR and p70S6 kinase suppress OPN-induced NF-κB and AP-1 mediated ICAM-1 expression**. **A**. MCF-7 cells were transfected with wild type or rapamycin resistant mTOR or pretreated with 20 nM rapamycin for 1 h and then treated with 0.5 μM OPN for 24 h. **B**. In another experiments, MCF-7 cells were transfected with wild type or rapamycin resistant p70S6 kinase or pretreated with 20 nM rapamycin and then treated with OPN under the same condition as mentioned above. Cell lysates were analyzed by western blot using anti-ICAM-1 antibody. Actin was used as loading control. **C**.MCF-7 cells were transfected with ICAM-1 luciferase reporter construct along with Renilla luciferase and then treated with 0.5 μM OPN for 24 h or pretreated with rapamycin (20 nM) for 1 h and then treated with OPN. The transfection efficiency was normalized and fold changes in luciferase activity with respect to control were calculated, and mean ± S.D. of triplicate determinations are plotted. The values were also analyzed by One Way ANOVA (*, p > 0.05; **, p > 0.05). **D**. MCF-7 cells were individually transfected with IκBα sup. rep., wild type and dominant negative c-Jun or A-Fos and then treated with 0.5 μM OPN for 24 h. Cell lysates were analyzed by western blot using anti-ICAM-1 antibody. Actin was used as loading control.

To examine the role of mTOR signaling in OPN-induced ICAM-1 expression; MCF-7 cells were individually transfected with wild type or rapamycin resistant mTOR or pretreated with rapamycin and then treated with OPN. Cell lysates were analyzed by western blot using anti-ICAM-1 antibody. The results indicated that overexpression of wt or rapamycin resistant mTOR inhibits whereas rapamycin enhances OPN-induced ICAM-1 expression suggesting that mTOR is involved in this process (Fig. 2A). To investigate the role of p70S6 kinase in OPN-induced ICAM-1 expression, cells were transfected with wild type or rapamycin resistant p70S6 kinase or pretreated with rapamycin and then treated with OPN. The cell lysates were analyzed by western blot using anti-ICAM-1 antibody and the data shown that overexpression of wt or rapamycin resistant p70S6 kinase attenuates whereas rapamycin augments OPN-induced ICAM-1 expression indicating that p70S6 kinase plays important role in this process (Fig. 2B).

To further study the role of mTOR/p70S6 kinase on ICAM-1 transcriptional activity in response to OPN; cells were transiently transfected with ICAM-1 luciferase reporter construct. Transfected cells were treated with rapamycin and then with OPN. The transfection efficiency was normalized by cotransfecting the cells with Renilla luciferase vector. Changes in luciferase activity with respect to control were calculated. The results indicated that OPN induces ICAM-1 transcriptional activity and rapamycin augments ICAM-1 transcription in response to OPN (Fig. 2C). To assess the role of NF-κB and AP-1 in OPN-induced ICAM-1 expression, MCF-7 cells were individually transfected with IκBα super repressor, wt and dominant negative c-Jun, and A-Fos and then treated with OPN. Cell lysates were analyzed by western blot using anti-ICAM-1 antibody. The results indicated that IκBα super repressor, dominant negative c-Jun and A-Fos suppressed whereas wt c-Jun enhanced OPN-induced ICAM-1 expression (Fig. 2D). Actin was used as loading control.

### mTOR plays crucial role in OPN-induced NF-κB activation

**Figure 3:**
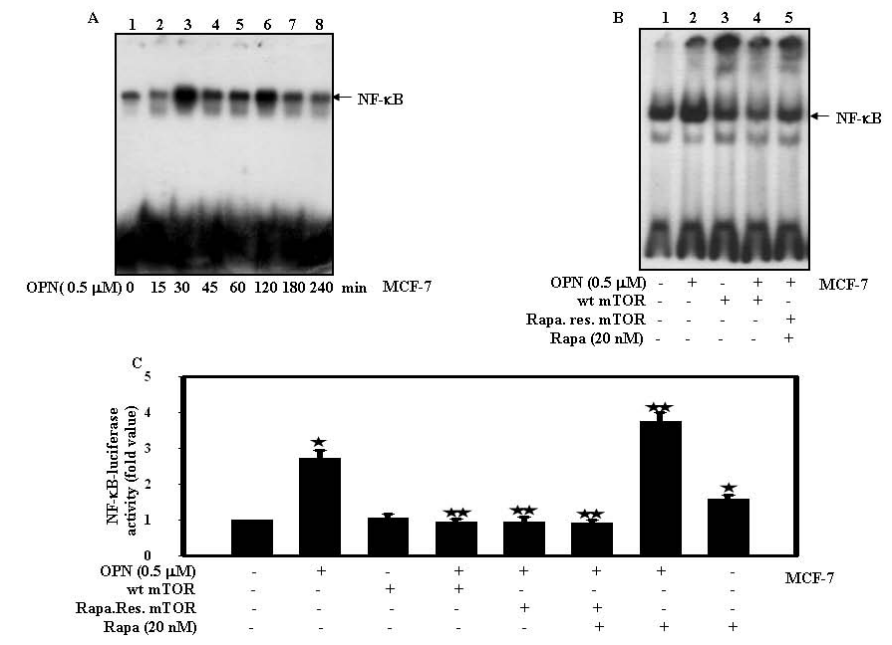
**mTOR suppresses OPN-induced NF-κB Activation**. **A and B**. MCF-7 cells were treated with 0.5 μM OPN for 0-240 min or transiently transfected with wild type or rapamycin resistant mTOR and then treated with OPN. The non-transfected or transfected cells were treated with rapamycin alone or along with OPN under the same condition as described in Materials and Methods. Nuclear extracts were prepared and analyzed by EMSA. ***C***. MCF-7 cells were transfected with wt or rapamycin resistant mTOR along with NF-κB luciferase reporter construct and then treated with 0.5 μM OPN. The non-transfected or transfected cells were treated with rapamycin alone or along with OPN. The transfection efficiency was normalized by Renilla and fold changes in luciferase activity with respect to control were calculated, and mean ± S.D. of triplicate determinations are plotted. The values were also analyzed by One Way ANOVA (*, p < 0.05; **, p < 0.05).

To investigate the effect of OPN on NF-κB-DNA binding in a time dependent manner, MCF-7 cells were treated with OPN for 0-240 min; nuclear extracts were prepared and analyzed by EMSA. The data showed that OPN induces NF-κB-DNA binding in a time dependent manner, with maximum binding at 30 min (Fig. 3A). To examine the role of mTOR on OPN-induced NF-κB-DNA binding; cells were either transiently transfected with wt type mTOR or rapamycin resistant mTOR, treated with rapamycin and then with OPN. The data suggested that mTOR inhibits OPN-induced NF-κB-DNA binding (Fig. 3B). To elucidate the role of mTOR on OPN-induced NF-κB transcriptional activity; cells were either transiently transfected with wt type mTOR or rapamycin resistant mTOR along with NF-κB luciferase reporter construct (pNF-κB-Luc) or pretreated with rapamycin and then with OPN. Changes in luciferase activity with respect to control were calculated. The transfection efficiency was normalized by transfecting the cells with Renilla luciferase vector. The results indicated that the level of OPN-induced NF-κB transcriptional activity in mTOR transfected cells decreased as compared to cells treated with OPN alone or rapamycin along with OPN. The data suggested that overexpression of mTOR inhibits OPN-induced NF-κB transactivation (Fig. 3C).

### OPN-induced AP-1 activation is downregulated by mTOR

**Figure 4:**
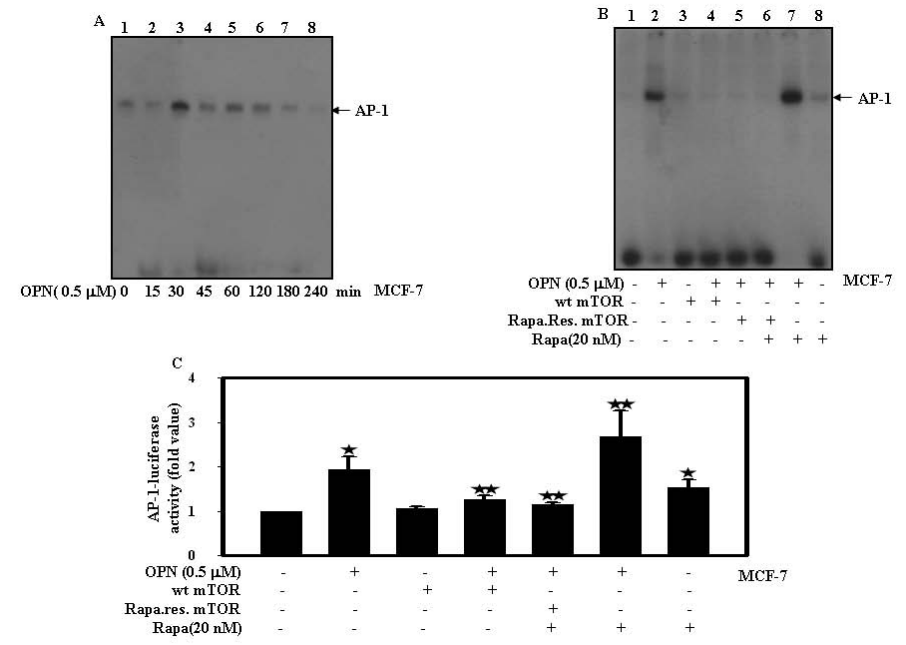
**mTOR down reguates OPN-induced AP-1 activation**. **A and B**. MCF-7 cells were treated with 0.5 μM OPN for 0-240 min or transiently transfected with wild type or rapamycin resistant mTOR and then treated with OPN. The non-transfected or transfected cells were treated with rapamycin alone or along with OPN. Nuclear extracts were prepared and analyzed by EMSA. ***C***. MCF-7 cells were transiently transfected with wild type or rapamycin resistant mTOR along with pAP-1-Luc. The non-transfected or transfected cells were treated with rapamycin alone or along with OPN. The transfection efficiency was normalized by Renilla and fold changes in luciferase activity with respect to control were calculated, and mean ± S.D. of triplicate determinations are plotted. The values were also analyzed by One Way ANOVA (*, p < 0.05; **, p < 0.05).

To check the effect of OPN on AP-1-DNA binding, MCF-7 cells were treated with OPN for 0-240 min; nuclear extracts were prepared and analyzed by EMSA. The data showed that OPN induces AP-1-DNA binding maximum at 30 min (Fig. 4A). To further examine the role of mTOR on AP-1-DNA binding; cells were either transiently transfected with wt mTOR or rapamycin resistant mTOR in absence or presence of rapamycin and then treated with OPN. The data suggested that mTOR inhibits OPN-induced AP-1-DNA binding (Fig. 4B). To elucidate the role of mTOR on OPN-induced AP-1 transcriptional activity; cells were either transiently transfected with wt mTOR along with AP-1 luciferase reporter construct (pAP-1-Luc) and then treated in absence or presence of OPN. In separate experiments, rapamycin resistant mTOR transfected cells were pretreated with rapamycin and then treated with or without OPN and changes in luciferase activity with respect to control were calculated. The transfection efficiency was normalized by transfecting the cells with Renilla luciferase vector. The results indicated that the level of AP-1 transcriptional activity in mTOR transfected cells decreased as compared to cells treated with OPN alone or rapamycin along with OPN (Fig. 4C). The data reveals that overexpression of mTOR inhibits OPN-induced AP-1 transactivation.

### OPN-induced cross talk between NF-κB and AP-1 is unidirectional towards AP-1

**Figure 5:**
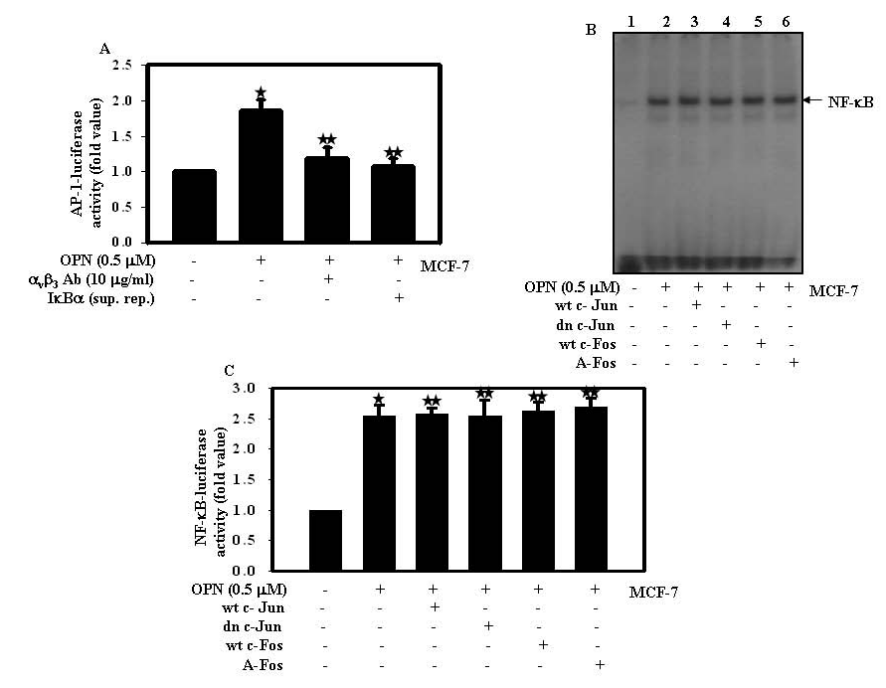
**NF-κB regulates OPN-induced AP-1 activation**. **A**. MCF-7 cells were transfected with pAP-1-Luc along with Renilla luciferase and then either treated with OPN alone or pretreated with anti-α_v_β_3 _integrin blocking antibody along with OPN. In separate experiments, cells were transfected with IκBα super-repressor along with pAP-1-Luc and then treated with OPN. The transfection efficiency was normalized by Renilla and fold changes in luciferase activity with respect to control were calculated, and mean ± S.D. of triplicate determinations are plotted. The values were also analyzed by One Way ANOVA (*, p < 0.05; **, p < 0.05). **B**. MCF-7 cells were individually transfected with wild type and dominant negative c-Jun, wild type c-Fos, A-Fos and then treated with OPN for 30 min. Nuclear extracts were prepared and analyzed by EMSA. **C**. MCF-7 cells were individually transfected with wild type and dominant negative c-Jun, wild type c-Fos, A-Fos along with pNF-κB-Luc and then treated with OPN for 24 h. The transfection efficiency was normalized by Renilla and fold changes in luciferase activity with respect to control were calculated, and mean ± S.D. of triplicate determinations are plotted. The values were also analyzed by One Way ANOVA (*, p < 0.05; **, p < 0.05).

To investigate the involvement of α_v_β_3 _integrin and NF-κB in OPN-induced AP-1 transcriptional activity, cells were transiently transfected with IκBα super repressor (IκBα sup. rep.) along with AP-1 luciferase reporter construct (pAP-1 Luc) and then treated with OPN. In separate experiments, AP-1-Luc transfected cells were pretreated with α_v_β_3 _integrin blocking antibody and then treated with OPN. The transfection efficiency was normalized by transfecting the cells with pRL vector and changes in luciferase activity with respect to control were calculated. The data indicates that α_v_β_3 _integrin blocking antibody or IκBα sup. rep. suppresses OPN-induced AP-1 transcriptional activity (Fig. 5A). To examine whether AP-1 is also involved in regulation of OPN induced NF-κB activation, cells were individually transfected with wt and dominant negative c-Jun, c-Fos or A-Fos and then treated with OPN and EMSA was performed. The results indicated that wt and dominant negative c-Jun, c-Fos and A-Fos had no effect on OPN-induced NF-κB-DNA binding (Fig. 5B). This was further confirmed by NF-κB luciferase assay (Fig. 5C) under the same conditions as described in Fig. 5B. The results revealed that AP-1 or its components have no effect on OPN-induced NF-κB activation (Fig. 5B and 5C) and further confirmed that OPN-induced NF-κB regulates AP-1 activation in a unidirectional manner.

### OPN induces phosphorylation of p70S6 kinase at Thr-421/Ser-424, but not at Thr-389 and Ser-371 and has no effect on mTOR phosphorylation at Ser-2448

**Figure 6:**
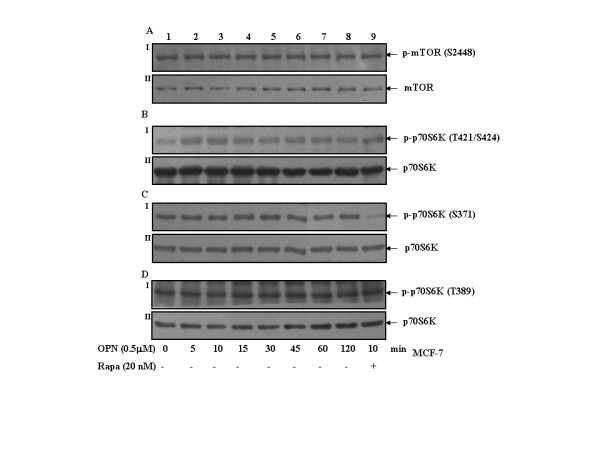
**OPN induces phosphorylation of p70S6 kinase at Thr-421/Ser-424, but not at Thr-389 and Ser-371 and has no effect on mTOR phosphorylation**. ***A-D***. MCF-7 cells were either treated with 0.5 μM OPN for 0-120 min or pretreated with 20 nM rapamycin for 1 h and then treated with 0.5 μM OPN for 10 min. Cell lysates containing equal amount of total proteins were resolved by SDS-PAGE and immunoblotted with anti-p-mTOR or anti-p-p70S6 kinase (Thr-421/Ser-424, Ser-371 or Thr-389) antibody. These blots were reprobed with anti-mTOR or anti-p70S6 kinase antibody.

To study the effect of OPN on phophorylation of mTOR and p70S6 kinase, MCF-7 cells were either treated with OPN for 0-120 min or pretreated with 20 nM rapamycin for 1 h and then treated with OPN for 10 min. The results indicated that OPN has no effect on mTOR phosphorylation at Ser-2448 and p70S6 kinase phosphorylation at Thr-389 and Ser-371, while it does induce p70S6 kinase phosphorylation at Thr-421/Ser-424. Rapamycin suppresses basal level phosphorylation of p70S6 kinase at Ser-371 but does not have any effect on Thr-389 and Thr-421/Ser-424 phosphorylation (Fig. 6, panels A-D).

### OPN induces mTOR independent p70S6 kinase phosphorylation at Thr-421/Ser-424 through MEK/ERK pathway

**Figure 7:**
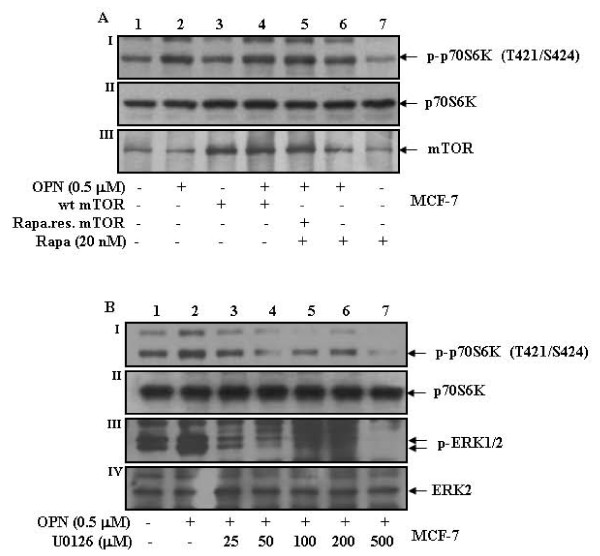
**OPN induces mTOR independent p70S6 kinase phosphorylation at Thr-421/Ser-424 through MEK/ERK pathway**. **A**. MCF-7 cells were transiently transfected with wild type or rapamycin resistant mTOR. After 48 h, cells were either treated with OPN or pretreated with rapamycin and then treated with 0.5 μM OPN for 10 min. Cell lysates containing equal amount of total proteins were analyzed by western blot with anti-p-p70S6 kinase (Thr-421/Ser-424) or anti-mTOR antibody. The blots were reprobed with anti-p70S6 kinase antibody. **B**. MCF-7 cells were pretreated with 0-500 μM U0126 (MEK inhibitor) for 1 h and then treated with 0.5 μM OPN for 10 min. Cell lysates were analyzed by western blot with anti-p-p70S6 kinase (Thr-421/Ser-424) or anti-p-ERK1/2 antibody. The same blots were reprobed with anti-p70S6 kinase or anti-ERK2 antibody.

To delineate the role of mTOR on p70S6 kinase phosphorylation at Thr-421/Ser-424, MCF-7 cells were either transiently transfected with wt or rapamycin resistant mTOR or pretreated with rapamycin (20 nM) for 1 h and then treated with OPN for 10 min. The results revealed that mTOR does not play any role in OPN-induced p70S6 kinase phosphorylation at Thr-421/Ser-424 (Fig. 7A). To examine the role of MEK/ERK on p70S6 kinase phosphorylation at Thr-421/Ser-424, cells were pretreated with MEK inhibitor, U0126, for 1 h and then treated with OPN for 10 min. The results indicated that U0126 inhibits OPN-induced p70S6 kinase phosphorylation at Thr-421/Ser-424 (Fig. 7B) suggesting that MEK/ERK pathway plays significant role in p70S6 kinase phosphorylation in response to OPN.

## Discussion

Recent reports demonstrated that both stroma and tumor derived OPN regulate breast tumor progression. OPN is a matrix associated ECM protein and it's over expression confers malignant transformation in a variety of tumorigenic cell lines [10]. OPN was found to be a metastasis associated protein in breast cancer. Rudland et al have reported that majority of the breast cancer patients showed significantly higher level of OPN expression than normal individuals [31]. The level of serum OPN in patients with breast, lung and prostate cancers is higher as compared to controls. The concentration of OPN required in controlling various cellular signaling events leading to tumor progression is varied significantly. Earlier reports have shown that nanomolar concentrations of OPN regulate cell adhesion and migration through PI 3-kinase-dependent Akt phosphorylation pathway in prostate cancer cells. However, other studies have indicated that micromolar concentrations of OPN are required to regulate tumor growth through PI 3-kinase dependent uPA secretion and MMP activation in various cancer cells. Therefore, different concentrations of OPN might regulate these cellular functions depending on the degree of posttranslational modification, the sources from which it is obtained and the nature of cell lines used [10,32]. Thus the role of OPN in various pathophysiological conditions, particularly in cancer, suggested that the variation in post-translational modification such as glycosylation, phosphorylation and sulfation generate the different functional forms that might alter its normal physiological functions.

Recently, Rosette et al. have reported that ICAM-1 is likely to play a major role in invasion of cancer cells leading to tumor growth and metastasis in breast cancer [29]. However, the mechanism by which OPN regulates ICAM-1 expression in breast cancer cells is not well defined. Here, we provide the experimental evidence indicating that OPN induces ICAM-1 expression in breast cancer, MCF-7 cells. We also examined the role of mTOR and its downstream molecule, p70S6 kinase, in OPN-induced ICAM-1 expression and the data suggest that overexpression of both mTOR and p70S6 kinase inhibit whereas rapamycin augments OPN-induced ICAM-1 expression in MCF-7 cells. The data revealed that OPN induces ICAM-1 expression through NF-κB and AP-1 mediated pathway. Moreover, the results showed that rapamycin augments OPN-induced ICAM-1 promoter activity in these cells. Furthermore, OPN induces NF-κB activation and overexpression of mTOR suppresses NF-κB activation in these cells. Earlier reports have shown that inhibition of mTOR by rapamycin induced NF-κB activity in response to thrombin in endothelial cells [23].

Our data also revealed that overexpression of mTOR suppresses OPN-induced AP-1 activation and rapamycin enhances this OPN-induced effect. We also showed that OPN regulates cross-talk between NF-κB and AP-1 that leads to ICAM-1 expression in breast cancer cells. Here we provide the experimental evidence that OPN induces AP-1-DNA binding and overexpression of IκBα super repressor suppresses OPN-induced AP-1 transactivation. Moreover, the OPN-induced NF-κB activation is not being controlled by AP-1. These data suggested that OPN-induced cross talk between NF-κB and AP-1 is unidirectional towards AP-1. Previous report indicated that OPN regulates cell migration, adhesion, invasion, proliferation and intracellular signaling by interacting with its receptor α_v_β_3 _integrin in various cell types ([10,33] and [34]). Our data also showed that α_v_β_3 _integrin blocking antibody suppresses OPN-induced AP-1 transcriptional activity in MCF-7 cells suggesting that OPN induces AP-1 transcriptional activation by interacting with its receptor α_v_β_3 _intergrin. Thus, OPN upon binding with α_v_β_3 _integrin induces AP-1 transcriptional activity through NF-κB mediated pathway indicating a cross talk between NF-κB and AP-1 which in turn regulates ICAM-1 expression. Recent reports indicated that several mTOR inhibitors are currently under evaluation in preclinical and clinical studies [35]. In this study, we have shown that inhibition of mTOR and its downstream target p70S6 kinase by rapamycin potentiate OPN-induced ICAM-1 expression. The data are consistent with the earlier report that inhibition of mTOR enhances thrombin-induced ICAM-1 expression by accelerating and stabilizing NF-κB activation in endothelial cells [23]. In our study, we have evaluated the role of OPN and rapamycin on phosphorylations of mTOR and p70S6 kinase and the data suggested that OPN does not phosphorylate mTOR at Ser-2448 and p70S6 kinase at Thr-389 and Ser-371, but at Thr-421/Ser-424 sites. However, rapamycin does not affect phosphorylation of mTOR at Ser-2448 and p70S6 kinase at Thr-389 and Thr-421/Ser-424 but it does inhibit basal level of phosphorylation of p70S6 kinase at Ser-371.

**Figure 8:**
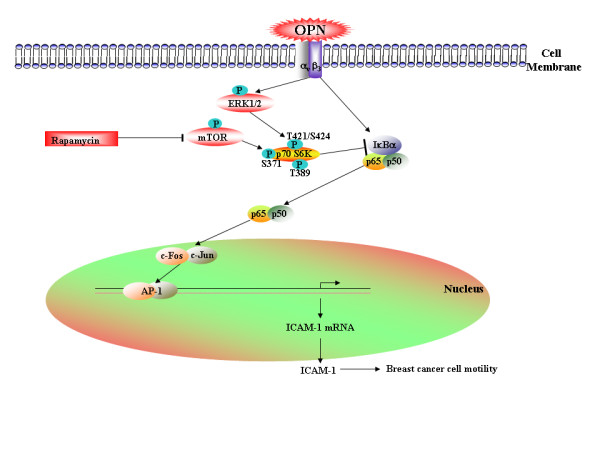
**Molecular mechanism of OPN-induced p70S6 kinase/mTOR regulated NF-κB/AP-1 mediated ICAM-1 expression in breast cancer cells**. OPN induces α_v_β_3 _integrin-mediated NF-κB/AP-1 dependent ICAM-1 expression. Overexpression of mTOR/p70S6 kinase suppresses OPN-induced ICAM-1 expression in breast cancer cells.

Phosphorylation of p70S6 kinase at Thr-421/Ser-424 exists in the autoinhibitory domain of carboxyl terminal, Thr-229 in activation loop, Thr-389 and Ser-371 in the linker domain, all of these are crucial for the activation of p70S6 kinase [17,18]. Earlier reports suggest that phosphorylation of p70S6 kinase at Thr-421/Ser-424 alone is not sufficient for the activation of p70S6 kinase [36]. But the phosphorylation of p70S6 kinase at Ser-371 is under the control of mTOR and is directly responsible for p70S6 kinase activation ([17,36] and [37]). Our study revealed that inhibition of mTOR activity by rapamycin suppresses basal level phosphorylation of p70S6 kinase at Ser-371 which may possibly be the reason for increased OPN-induced ICAM-1 expression and transactivation. Moreover, overexpression of mTOR and rapamycin have no effect on p70S6 kinase phosphorylation at Thr-421/Ser-424 which further confirmed that phosphorylation at this site is not responsible for the activation of p70S6 kinase. However, p70S6 kinase phosphorylation at Thr-421/Ser-424 site is being suppressed by MEK/ERK inhibitor, U0126. The data suggests that OPN-induced p70S6 kinase phosphorylation at Thr-421/Ser-424 site is not being controlled by mTOR; rather it is being regulated through MEK/ERK pathway. OPN has been reported as a diagnostic marker in patients with breast cancers [31] and suppression of tumor derived OPN by its antisense S-oligonucleotide and siRNA has been shown to suppress the *in vitro *proliferation, migration, and *in vivo *osteolytic metastasis in nude rats [38-40]. Thus, a better understanding of the molecular mechanism of regulation of ICAM-1 expression in response to OPN may help in developing a novel therapeutic approach for the treatment of breast cancer (Fig. 8).

## Conclusion

This study highlights the potential role of OPN to induce ICAM-1 expression through mTOR/p70S6 kinase pathway in breast cancer cells. The findings emphasize the importance of mTOR/p70S6 kinase pathway as a check point to regulate ICAM-1 expression in response to OPN. The data further revealed that OPN regulates cross talk between transcription factors NF-κB and AP-1 which is unidirectional towards AP-1 that in turn regulates ICAM-1 expression. Moreover, the results deciphered the role of OPN and rapamycin in regulating mTOR and p70S6 kinase phosphorylations and involvement of MEK/ERK pathway in this process. Breast cancer is one of the most debilitating diseases and earlier reports have shown that ICAM-1 plays important role in regulating invasion, tumor growth and metastasis in breast cancer. Therefore it is important to understand how OPN selectively regulate p70S6K/mTOR phosphorylation leading to NF-κB dependent AP-1 mediated ICAM-1 expression in breast cancer cells. Thus, the study suggests that blocking of OPN-induced ICAM-1 expression through mTOR/p70S6 kinase signaling may be an important therapeutic target for the management of breast cancer.

## Competing interests

The authors declare that they have no competing interests.

## Authors' contributions

MA designed and carried out all the experiments and wrote the initial drafts of the manuscript. GCK contributed to the conception and design of the entire study and the final editing of the manuscript. All authors read and approved the final manuscript.
